# An uncommon midfoot injury, naviculocuneiform and calcaneocuboid fracture dislocation: a case report

**DOI:** 10.1093/jscr/rjad113

**Published:** 2023-03-21

**Authors:** Fuminori Kamakura, Gaku Yasuda, Yoshimasa Ishigaki, Satoshi Goto

**Affiliations:** Department of Orthopedic Surgery, Fujimi-Kogen Hospital, Fujimi-Kogen Medical Center, Nagano, Japan; Department of Orthopedic Surgery, Fujimi-Kogen Hospital, Fujimi-Kogen Medical Center, Nagano, Japan; Department of Orthopedic Surgery, Fujimi-Kogen Hospital, Fujimi-Kogen Medical Center, Nagano, Japan; Department of Orthopedic Surgery, Fujimi-Kogen Hospital, Fujimi-Kogen Medical Center, Nagano, Japan

## Abstract

A 57-year-old man fell from a height of 6 m and injured his right foot. Imaging studies showed an uncommon injury; naviculocuneiform and calcaneocuboid joint fracture dislocations. He underwent a temporary fixation with Kirschner wires (K-wires), and the injured foot was immobilized with a below-knee splint. Weight-bearing was started gradually. The K-wires were removed at 8 weeks. Full weight-bearing was achieved 14 weeks after the operation. At 19 weeks, he returned to his carpentry job. At 1 year, he had no marked limitation of daily activities. Early recognition of these injuries is required to prevent persistent foot pain and long-term dysfunction.

## INTRODUCTION

Dislocations and fractures of midfoot joints, including the Chopart joint, are relatively rare injuries, with few reported cases [1, 2]. We encountered an uncommon case of fracture dislocation of the midfoot involving the naviculocuneiform and calcaneocuboid joints, similar to a Chopart injury. We present this rare injury with a review of the literature.

## CASE REPORT

**Figure 1:**
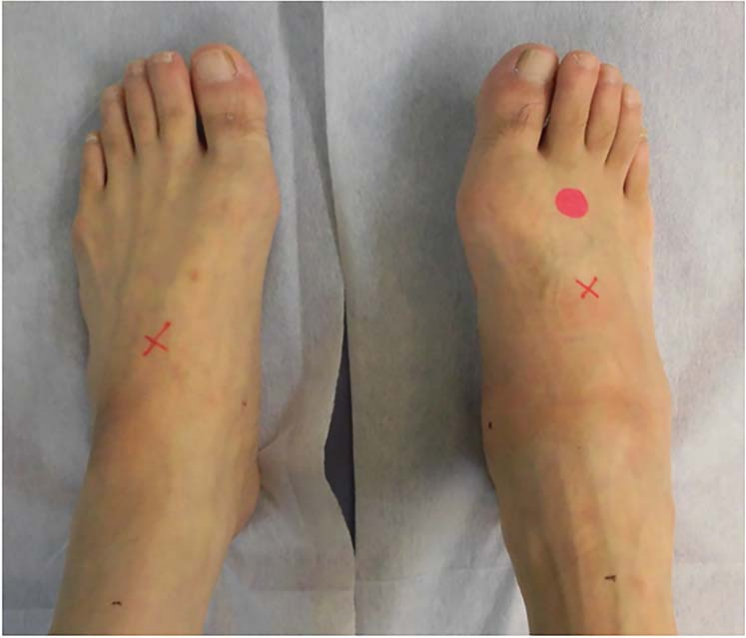
Physical examination shows swelling and deformity of the right foot.

**Figure 2:**
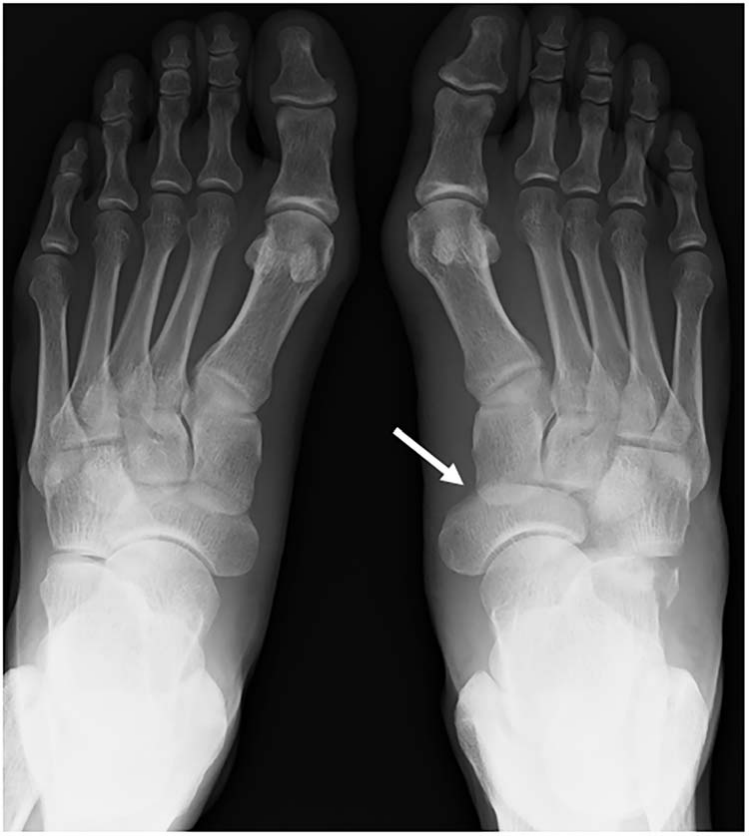
A plain radiograph shows lateral dislocation of the naviculocuneiform and calcaneocuboid joints of the right foot (arrow). The difference between the two sides is obvious.

**Figure 3:**
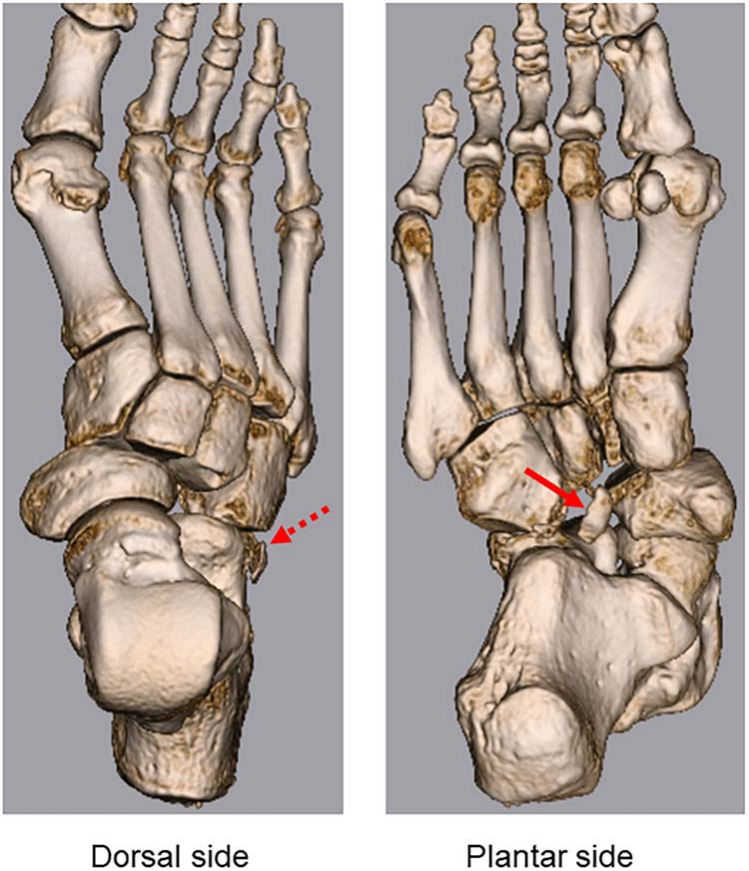
3D CT shows lateral dislocation of the naviculocuneiform and calcaneocuboid joints of the right foot, with fractures of the anterior edge of the calcaneus (broken arrow) and medial edge of the cuboid (arrow).

**Figure 4:**
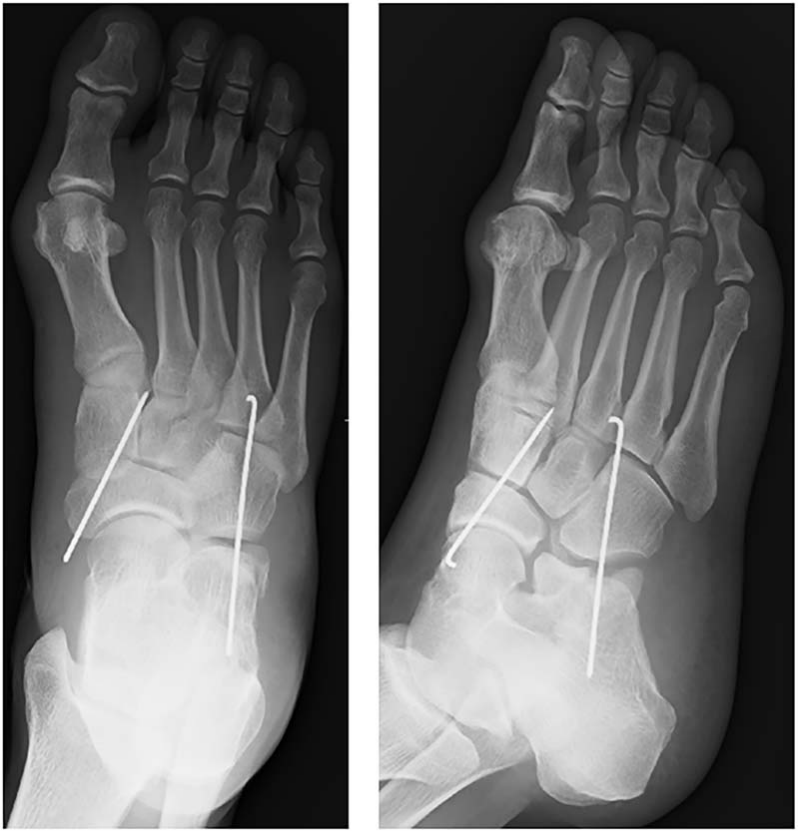
After fixation with K-wires. The dislocated site is reduced and stabilized.

**Figure 5:**
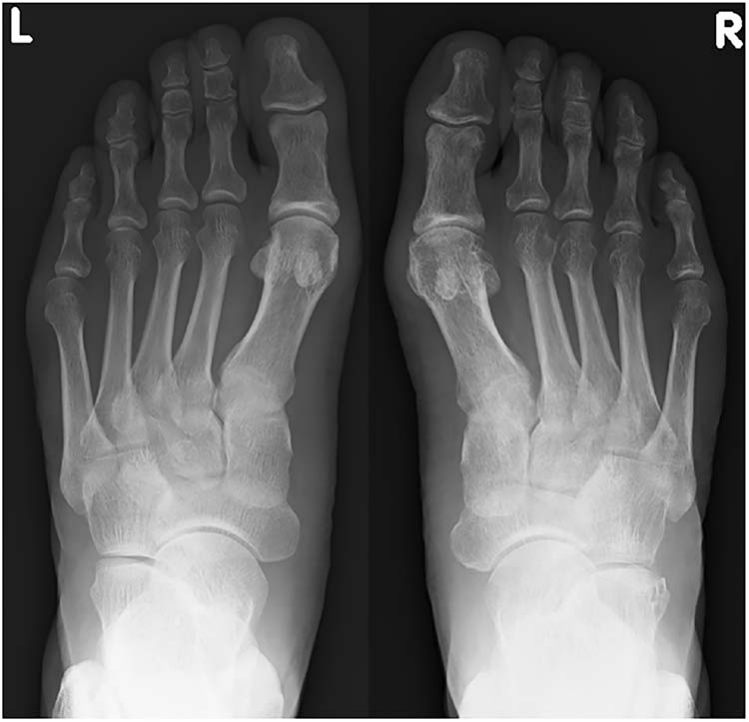
Plain radiograph at 1 year after the initial operation. There was no marked malalignment in the right foot.

A 57-year-old carpenter fell from a height of 6 m and hit the right half of his body. He visited the emergency department of our hospital the same day. Physical examination revealed tenderness, swelling and deformity in the right foot (Fig. 1). Plain radiography, computed tomography (CT) and three-dimensional (3D) CT showed an uncommon injury; lateral dislocation of the naviculocuneiform joint, lateral dislocation of the calcaneocuboid joint, compression fracture of the calcaneus and avulsion fracture of the cuboid in the right foot (Figs 2 and 3). The next day, we attempted closed reduction, but the reduction was unstable. Percutaneous fixation with Kirschner wires (K-wires) was performed to stabilize the dislocated fragments. Two 1.5-mm K-wires were inserted from the navicular and cuboid bones to the medial cuneiform and calcaneus bones, respectively, and the dislocated joints were stabilized (Fig. 4). Postoperatively, the foot was immobilized with a below-knee splint. Mild ankle exercise was initiated in the early postoperative phase. The K-wires were removed at 8 weeks. Full weight-bearing was started at 14 weeks, and he was able to walk without pain. At 19 weeks, he returned to his job. At 1 year after the operation, the American Orthopaedic Foot and Ankle Society midfoot score was 75/100 points. He had mild and occasional pain, but he could walk about 2000 steps consecutively with no support or difficulty, and he had no marked limitation of daily activities. Fracture dislocation sites were stable on plain radiography (Fig. 5).

We obtained patient consent for the publication of this article.

## DISCUSSION

Naviculocuneiform and calcaneocuboid fracture dislocations are extremely rare. Main and Jowett [1] evaluated 71 cases of midtarsal injuries and reported that midfoot injury was relatively rare. Furthermore, there are few case reports on naviculocuneiform and calcaneocuboid dislocations or fracture dislocations [3]. In these cases, although the injuries resembled Chopart joint injury, the talonavicular joint, a constituent of the Chopart joint, was intact.

The midfoot is composed of the navicular, cuboid and three cuneiform bones that lie between the hindfoot and forefoot and are vital to the stability of the transverse arch and longitudinal columns of the foot [3]. The bones are connected by strong ligaments. The joints of the navicular and three cuneiform bones are rigidly connected to the ligaments and common synovial capsule [4].

There are three functional units in the midfoot: the medial, middle and lateral columns [5]. These columns stabilize the midfoot while allowing functional movement. The anatomical features of the midfoot, including the uniquely shaped bones; strong ligaments and secondary support by tendons, muscles and other connective tissues; and rigidity of the three columns, provide the midfoot joint with advanced stability.

Midtarsal injuries tend to occur following high-energy trauma, namely, a traffic accident or fall from a height [4]. Previous studies have reported that these injuries were frequently overlooked or misdiagnosed [1]. Midfoot injury can negatively influence social aspects such as work and recreational activities. Therefore, early diagnosis and treatment of these injuries are required to avoid instability, long-term functional problems, foot pain and severe complications. History of the injury and physical examination is important for diagnosis. The most common symptom is severe pain; additionally, foot deformity can be masked by marked localized swelling on physical examination [4]. Since the injury tends to occur as a result of high-energy trauma, damage to other parts of the body should also be considered. In addition to plain radiography, CT is useful in diagnosing injuries; 3D CT provides more detailed images to diagnose small fractures and bone fragments [4]. In the present case, small fractures of the medial edge of the cuboid and anterior edge of the calcaneus were diagnosed using 3D CT (Fig. 3).

Chopart injury, which resembles the present case, is described as a relatively rare injury, with a reported incidence of 2.2/100 000 person-years [2]. Axial forces typically cause damage to the talonavicular and the calcaneocuboid joints [4]. This leads to a collapse of the medial and lateral longitudinal columns of the foot. In our patient, the mechanism of injury was guessed to be similar to that in Chopart injury.

Regarding treatment, a closed reduction should be attempted initially. If instability persists, temporary joint fixation using K-wires, screws, plates or external fixators may be considered. Primary arthrodesis sacrifices joint function and should be reserved for patients with severe cartilage damage [6]. However, cases of malunion might require corrective fusion [6]. In our case, closed reduction and temporary fixation with K-wires were performed. Ankle joint exercise initiated in the early postoperative stage aided in maintaining the range of articular motion and muscle strength. After the removal of the K-wires, the patient was permitted full weight-bearing gradually. No postoperative complications were noted during the recovery period.

The complication rate increases with delayed treatment. Compartment syndrome, a severe complication in the acute stage, requires immediate treatment to avoid irreversible damage and poor functional prognosis. Global deformity and instability of the foot will persist if reduction and suitable fixation fail. Appropriate reconstruction of the collapsed columns is important [3]. Avascular necrosis and nonunion can occur as a result of local vascular insufficiency, particularly in patients with a history of diabetes mellitus or smoking [7]. Posttraumatic arthritis also tends to occur when treatment is delayed [6]. Thus, early anatomical reduction and stable fixation are important to avoid arthritic deformity [6]. All of these complications can cause persistent foot pain and dysfunction. Preventing them is important when treating a midfoot injury.

In conclusion, we have reported an uncommon case of midfoot injury, with naviculocuneiform fracture and calcaneocuboid fracture dislocation. Early recognition of these injuries is required to prevent persistent foot pain and long-term dysfunction.

## CONFLICT OF INTEREST STATEMENT

None declared.

## FUNDING

The authors received no specific grant from any funding agency.

## DATA AVAILABILITY

All data found about this case are included in this published article.
